# Exploring Machine Learning Algorithms to Predict Diarrhea Disease and Identify its Determinants among Under-Five Years Children in East Africa

**DOI:** 10.1007/s44197-024-00259-9

**Published:** 2024-07-29

**Authors:** Tirualem Zeleke Yehuala, Nebiyu Mekonnen Derseh, Makda Fekadie Tewelgne, Sisay Maru Wubante

**Affiliations:** 1 Department Health informatics, Institute of Public Health, College of Medicine and Health Sciences, University of Gondar, Gondar, Ethiopia; 2 Department of Epidemiology and Biostatistics, Institute of Public Health, College of Medicine and Health Sciences, University of Gondar, Gondar, Ethiopia

**Keywords:** Prediction, Diarrheal disease, Machine learning, East Africa

## Abstract

**Background:**

The second most common cause of death for children under five is diarrhea. Early Predicting diarrhea disease and identify its determinants (factors) using an advanced machine learning model is the most effective way to save the lives of children. Hence, this study aimed to predict diarrheal diseases, identify their determinants, and generate some rules using machine learning models.

**Methods:**

The study used secondary data from the 12 east African countries for DHS dataset analysis using Python. Machine learning techniques such as Random Forest, Decision Tree (DT), K-Nearest Neighbor, Logistic Regression (LR), wrapper feature selection and SHAP values are used for identify determinants.

**Result:**

The final experimentation results indicated the random forest model performed the best to predict diarrhea disease with an accuracy of 86.5%, precision of 89%, F-measure of 86%, AUC curve of 92%, and recall of 82%. Important predictors’ identified age, countries, wealth status, mother’s educational status, mother’s age, source of drinking water, number of under-five children immunization status, media exposure, timing of breast feeding, mother’s working status, types of toilet, and twin status were associated with a higher predicted probability of diarrhea disease.

**Conclusion:**

According to this study, child caregivers are fully aware of sanitation and feeding their children, and moms are educated, which can reduce child mortality by diarrhea in children in east Africa. This leads to a recommendation for policy direction to reduce infant mortality in East Africa.

## Introduction

The major symptom of diarrhea is watery, loose, and more frequent bowel motions. It is caused by viruses, bacteria, parasites, medications, and lactose intolerance, among other possible causes [1]. According to a World Health Organization report, the second most common cause of death for children under five is diarrhea. Globally, there are nearly 1.7 billion child disease cases of diarrhea every year, and diarrhea kills about 525,000 children under five per year. Diarrhea is the cause of almost 1.6 million child fatalities annually, or nearly one in every five child deaths [2]. More than 90% of children under the age of five die from diarrhea in low- and lower-middle-income countries in 2021; in South Asia and sub-Saharan Africa (SSA), this percentage rises to 88% [3]. Diarrhea poses numerous challenges for children, such as decreased appetite, vomiting, low electrolyte levels, abdominal pain, undernourishment, heightened vulnerability to other contagious illnesses, and postponed physical and mental maturation, perhaps leading to impairment [3, 4].

Several studies have shown that East African nations have a significant prevalence of diarrheal illnesses in children under the age of five. The prevalence of diarrheal illness in Ethiopia is 8.5–30.5% [5], in Nairobi 25.6% [6], in Uganda 7.1% [7], in north Sudan, south kordofan, blue Nile 20%,40%,19 respectively [8],and in Rwanda 12.7% children’s had diarrhea [9]. Other research in Malawi, Rwanda, and Uganda found that the prevalence of diarrheal illnesses was 20% [10], 26.7% [11], and 32% [12], respectively. It is possible to prevent diarrheal illness by using clean, safe drinking water, practicing good hygiene, and hand washing correctly, which helps stop the spread [13]. However, it is challenging in the world’s poorest nations, such as Ethiopia, Sudan, and Somalia [14].

Several studies are involved on diarrhea disease among children under five years old through the application of classical statistical analysis techniques, which could limit the potential to discover hidden knowledge. No review exists focusing on early prediction of diarrhea disease by the use of machine learning. The study have been conducted in Ethiopia, Senegal, North Tanzania, Kenya, Uganda, sub- Saharan [15–20].

Little is known by using cross-sectional studies, unmatched case-control studies, retrospective cross-sectional studies, multivariate logistic regression, and community-based longitudinal study analysis. To the best of our knowledge, no prior researcher has attempted to use machine learning approaches to predict diarrhea in children under five. Therefore, the goal of this research is to use machine learning algorithms to determine the factors or determinants that influence diarrhea in children under five and to create prediction models based on those factors. In conclusion, the two primary questions this study seeks to address are as follows:

**RQ1**

Which determinants are the most significant for diarrhea disease?

**RQ2**

Which machine learning models help to effectively predict a diarrhea disease?

## Method and Materials

In this section, the 12 East Africa DHS data sets were used. The prediction models for diarrhea disease based on supervised machine learning algorithms using random forest, logistic regression, decision trees, and gradient boosting algorithms are presented. Lastly, the evaluation methods of the supervised machine learning model will be discussed in detail.

### Study Setting

This study was conducted in East African countries using the DHS data set. Geographically, east Africa is a sub-region of Africa that includes 20 internationally known countries, among which Burundi, Ethiopia, Comoros, Uganda, Rwanda, Tanzania, Mozambique, Madagascar, Zimbabwe, Kenya, Zambia, and Malawi were the Eastern African nations included in this study.

### Data Source

The Measure DHS program served as the study’s data source. To access it, go to http://www.dhsprogram.com and submit the project title and study justification as part of a request. DHS data is a home survey that is nationally representative and is gathered on a regular basis from different groups. We used the Kids Record dataset (KR file) for this investigation. The most recent population health surveys from 12 countries in Eastern Africa were used in this study. Due to their lack of DHS conduction experience and the length of time since their last DHS conduction, the other East African counters were excluded.

### Population, and Eligibility Criteria

The study populations in this study were all under-five children who were in the designated enumeration regions during the time of DHS data collection, whereas all under-five children in east African countries aged 0 to 59 months were regarded as the source population.

### Study Variables and Measurements

In this investigation, the outcome variables were binary: when asked if they had diarrhea in the previous two weeks, those mothers who said that they had were coded as 1, and those who responded no were coded as 0. The features (independent variables) we will select by using feature selection methods.

### Sample Size Determination and Sampling Technique

We used a weighted sample of 89,875 children aged 0 to 59 months across 12 east African countries using the recent DHS dataset. A two-stage stratified cluster sampling technique was used to select study participants. For this study, we used the Kids Record dataset (KR file). The two-stage stratified sampling approach was used as the basis for the procedure. Enumeration Areas (EAs) were first chosen at random in accordance with their respective clusters. Households were chosen for the second stage. In each selected household, mothers were interviewed with an individual questionnaire.

### Data Analysis Procedure

**Fig. 1:**
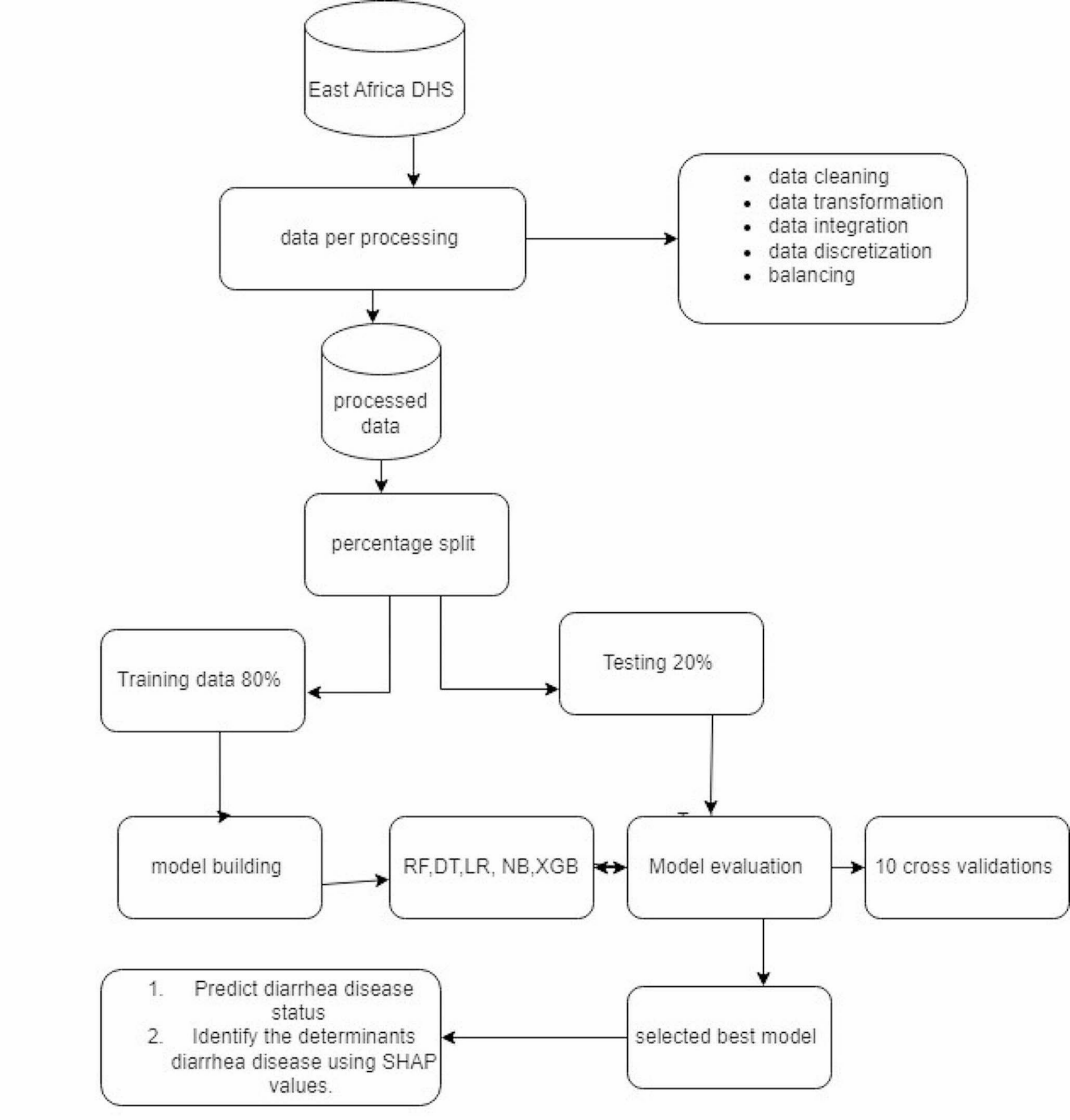
Overall data preparation and analysis process for predicting diarrhea disease status

A demographic and health survey data set from 12 East African countries was utilized in this study to predict diarrhea disease. Machine learning algorithms were used to come up with objective predictions about diarrhea disease and to identify the determinants that lead children’s to have diarrhea disease. By using machine learning algorithms and training data sets, we developed the diarrhea disease predictive model. Before we build a predictive model, we perform data processing. Data processing is a machine learning technique that transforms raw data into an understandable format [21]. Data processing and analysis are performed using Python (version 3.9) software and some basic packages like Panda, Scikit-Learn, Imblearn, Theano, Numpy, and Seaborne or Matplotlib, which are utilized for preparing data, discretizing data, transforming data, and choosing, visualizing, exploring, training, and evaluating models. Finally, we develop a predictive model that predicts both the diarrheal status and its associated determinants (Fig. 1).

### Data PreProcessing

#### Data Cleaning

In this study, data processing and analysis will be performed using Python software. The DHS data set by nature is not clean and aggregated; it needs different tasks like data cleaning, data transformation, exploratory data collection, data integration, normalization, dimensionality reduction, data discretization, model selection, model training, and model evaluation. Preprocessing data has several benefits, including raising the model’s accuracy and positively affecting the model’s performance [22]. To address noise, missing values, and outliers, we used data cleaning. It is impossible to send raw data through a model testing and training process since it is always missing and it creates biased or misinformed [23]. We evaluated them to ensure that outliers were present. However, missing values were found for some features, ranging from 0.5 to 3% of the dataset. We used mean and most frequent value (mode) imputing methodologies to address these missing values for the continuous and categorical variables, respectively.

#### Feature Selection

In this study, there are many features in the dataset, including over a thousand features. However, not all of these features are always relevant; therefore, feature selection is essential since unnecessary features during model training cause us to degrade the model’s overall accuracy, increase its complexity, limit its capacity to be generalized, and bias the model [24]. We used wrapper feature selection-based machine learning algorithms and SHAP values to select relevant features for model building.

#### Data Transformation

In data transformation, due to the non-aggregate nature of the DHS dataset, it is necessary to reorganize or restructure the raw data and convert the data to create the same data types [25]. This is important for machine learning to retrieve strategic information efficiently and easily. In this study, we utilized one-hot-encoding techniques implemented in Python to encode categorical to dummy variables, with each category as a separate variable coded as 0 or 1 to indicate mothers who had no diarrhea in the previous two weeks and had diarrhea in the previous two weeks, respectively.

#### Data Discretization and Integration

The technique of discretization allows us to convert continuous variables into a discrete form [26]. In order to make the data easier to grasp and analyze, we did data discretization: we converted continuous variables into discrete features according to DHS guidelines to minimize outlier influence and reduce noise. In this study, continuous features like children’s age are categorized into intervals or ranges of 0–6 months, 7–12 months, 13–23 months, and 24–59 months for easy analysis and interpretation. Additionally, in this study, we integrate 12 country datasets into a single dataset.

#### Class Balancing

When presented with an uneven data set, machine learning algorithms are prone to bias toward the majority class [27]. We balanced before training the prediction model for resampling imbalanced datasets; SMOTE oversampling was used to improve classification performance. In this study, we extracted 89,875 records from these 76,672 (85%) children who have no diarrhea disease and 13,203 (15%) who have diarrhea disease.

### Machine Learning Classifiers

The study used logistic regression, gradient boosting, random forest, K-nearest neighbor, and decision tree classifiers to predict diarrhea in children under five. Logistic regression is used to determine the likelihood that an event will succeed or fail. When the target variable has a binary (yes/no) nature, it is utilized [28]. Logistic regression is a very effective training method that is simpler to apply and analyze. In addition to logistic regression, the most widely used method for representing predictions is the decision tree. Decision trees are resistant to outliers and can be fairly strong when employed in ensemble algorithms since they are clearly interpretable and can handle huge, complex datasets efficiently without imposing a complex parametric structure [29]. The random forest algorithm is a type of supervised classification method where several decision trees cooperate with one another. The class that receives the most votes is the one that our model predicts. The decision tree algorithm’s drawbacks are removed as every tree in the random forest predicts a class [30]. As a result, the dataset becomes less overfat and more accurate. If a sizable percentage of record values are missing, the random forest approach may still yield the same results when applied to huge datasets.

#### Evaluation Criteria

A confusion matrix was utilized to study the model performance, and a number of common evaluation metrics, such as accuracy score, ROC curve, precision (P), recall (R), and F-measure, were used to assess the performances of our prediction models. These are their succinct descriptions: A table that makes it possible to see how well a supervised learning algorithm is performing is called a confusion matrix. Children who are accurately diagnosed and anticipated to have diarrhea are referred to as true positives (TP). When a model accurately predicts that the children did not develop diarrhea, it is referred to as a true negative (TN). Models that predict children having diarrhea inaccurately are known as false positives (FP). False negatives (TN) are samples that have been mistakenly classified as being free of diarrhea.

Furthermore, the Receiver Operating Characteristics Curve, often known as the ROC curve, provides a comprehensive assessment of a model’s accuracy and filters the range of threshold values for decision-making [31]. In a test dataset, the binary classifier predicts each data instance as either positive or negative. Positive and negative classes’ 2 × 2 matrices are shown in the following table. The model’s effectiveness is measured by the confusion matrix (Table 1).

**Table 1:** Confusion matrix

N = Number of instances		**Diarrheal disease**	
Predicted by test		Yes	No
	Yes	TP (yes)	FP (Type 1 error)
	No	FN (Type 2 error)	TN(no)

Precision = (TP)/(TP + FP)

Recall = (TP)/(TP + FN)

F–Measure = (2 * Precision * Recall)/(Precision + Recall)

Accuracy = ((TP + TN)/(TP + TN + FP + FN)) × 100

## Results

### Description of Diarrhea Disease in East African

This study investigated a sample of 89,875 children under the age of five from 12 countries in East Africa that were part of a demographic and health survey. Overall, it was shown that 15% of children had diarrhea, and 85% of children did not have diarrhea. Most of the records in this study are for 50,262 (56%) of 25- to 59-month-old children. In the age range of 7–12 months, 27.3% of children had diarrhea, compared to 25–59 months. 10% of children had diarrhea. And my mother has no education. 16% of children had diarrhea due to their mothers having primary education. 13% of the children had diarrhea, so the mother is educated and has a history of diarrhea. Approximately 15% of children who used unimproved toilets had diarrhea, compared to 14% who used improved toilets. Compared to mothers who experienced media exposure, 86% of children had not diarrhea show (Table 2).

**Table 2:** Description of diarrhea disease in East African

**Variable**	**Diarrheal**	
**Child age**	**Yes**	**No**
0-6 month	1290(11.43%)	10001(88.57%)
7-12 month	2693(27.3%)	7160(72.7%)
13-23 month	4405(24%)	14064(76%)
24-59 month	4815(10%)	45447(90%)
**Education**		
Non	6905(16%)	20927(87%)
Primary	3163(13%)	37363(84%)
Secondary	3135(15%)	18382(85%)
**Twin status**		
No	12,852(15%)	74,556(85%)
Yes	351(14%)	2,116 (86%)
**Sources of toilets**		
unimproved toilet	8,961(15%)	50,396(85%)
improved toilet	4,241(14%)	26,268(86%)
**Working status**		
Not working	7,883(15%)	44,455(84%)
Working	3,785(14%)	23,697(86%)
**Number of children**		
One child	5,164(16%)	27,699(84%)
Greater than two child	8,039(14%)	48,973(86%)
**Sources of drinking water**		
unimproved water	4,665(15%)	26,072(85%)
improved water	8,538(14%)	50,600 (86%)
**Media exposure**		
No	8, 69(15%)	49,277(85%)
Yes	4,509(14%)	27,395(86%)
**Immunization status**		
No vaccinated	255(10%)	2,113(90%)
Partial vaccinated	5,949(17%)	29,051(83%)
Fully vaccinated	6,999(13%)	45,508(87%)

### Class Balancing

**Fig. 2:**
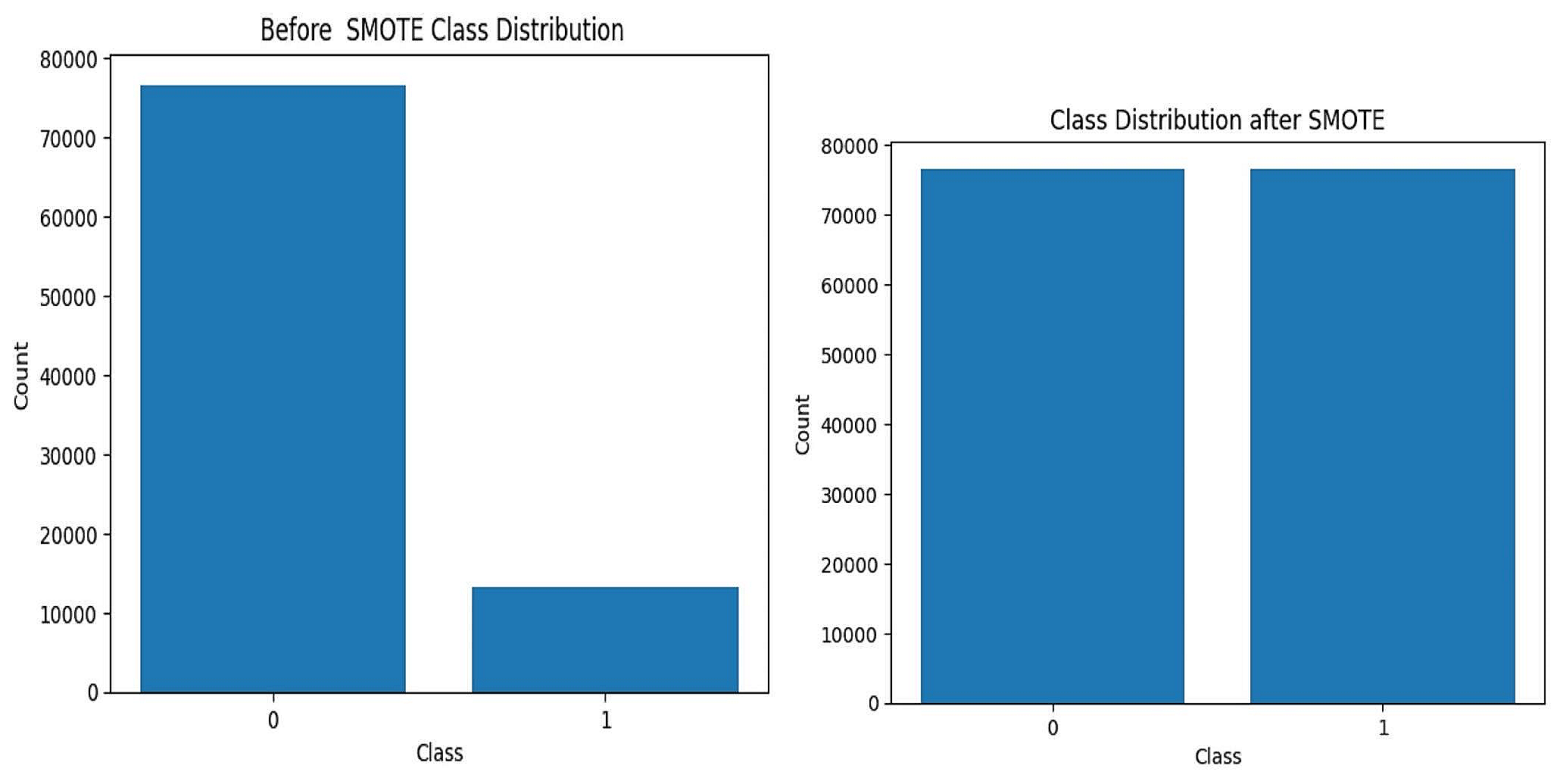
Before and after applying SMOTE balancing

In order to create balancing data for this study, we used the Synthetic Minority Oversampling Technique (SMOTE). This technique generates additional synthetic observations from the minority category in order to balance the unequal distribution of the outcome variable. Before smote balancing, having no diarrhea disease was 13,203 (15%), and having diarrhea was 76,672 (85%). We obtained a balanced sample of people who had diarrhea disease with counts of label 76,672 and not with counts of label 76,672 (Fig. 2).

### Determinant Selection /Features Selection

**Fig. 3:**
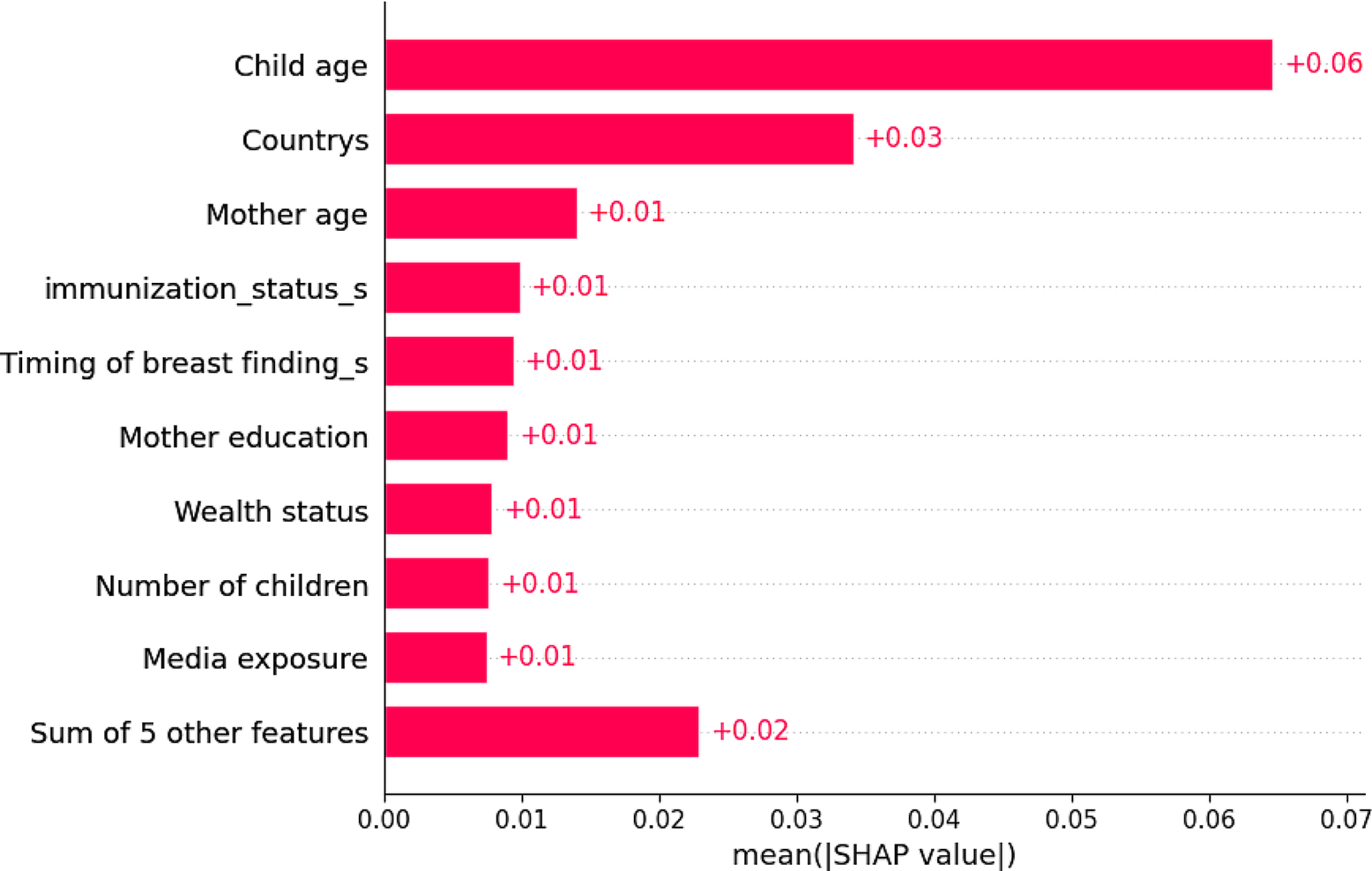
Important determinants selected by random forest mode

Important features Selection was a technique for identifying important and essential subsets of features because it improved learning performance, helped select and impact determinants by eliminating extraneous or excessive features, and cut down on training time [30]. In this study, we used recursive feature elimination (RFE) to infer features’ relevance using an estimate of their importance from a random forest model, and all features were selected. We used a random forest with SHAP values to narrow down the set of potential features shown in Fig. 3.

### ML Classifier Results

In order to identify determinants and predict diarrhea disease among under-five children using machine-learning techniques, supervised machine learning algorithms were considered, such as Decision Tree (DT), GB (Gradient Boosting), K-Nearest Neighbor, Logistic Regression (LR), and Random Forest (RF). The data set was divided into 80 for training and 20% for test sets. Python software version 3.9, which computes machine-learning algorithms (ML), was utilized for analysis.

**Fig. 4:**
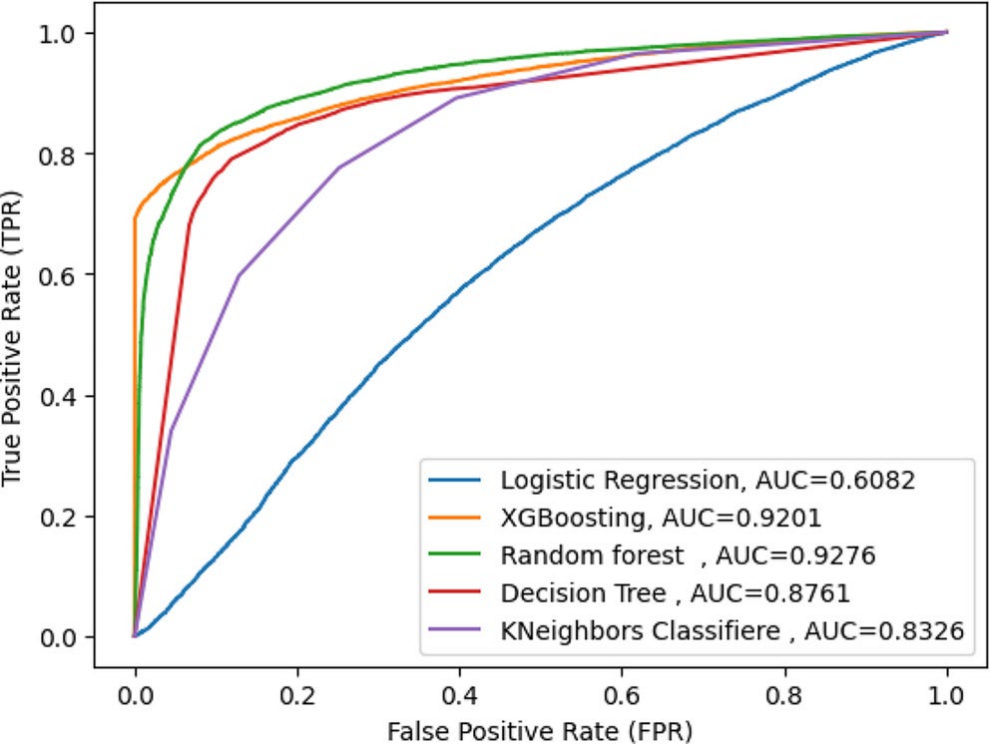
AUC curve for proposed model

**Fig. 5:**
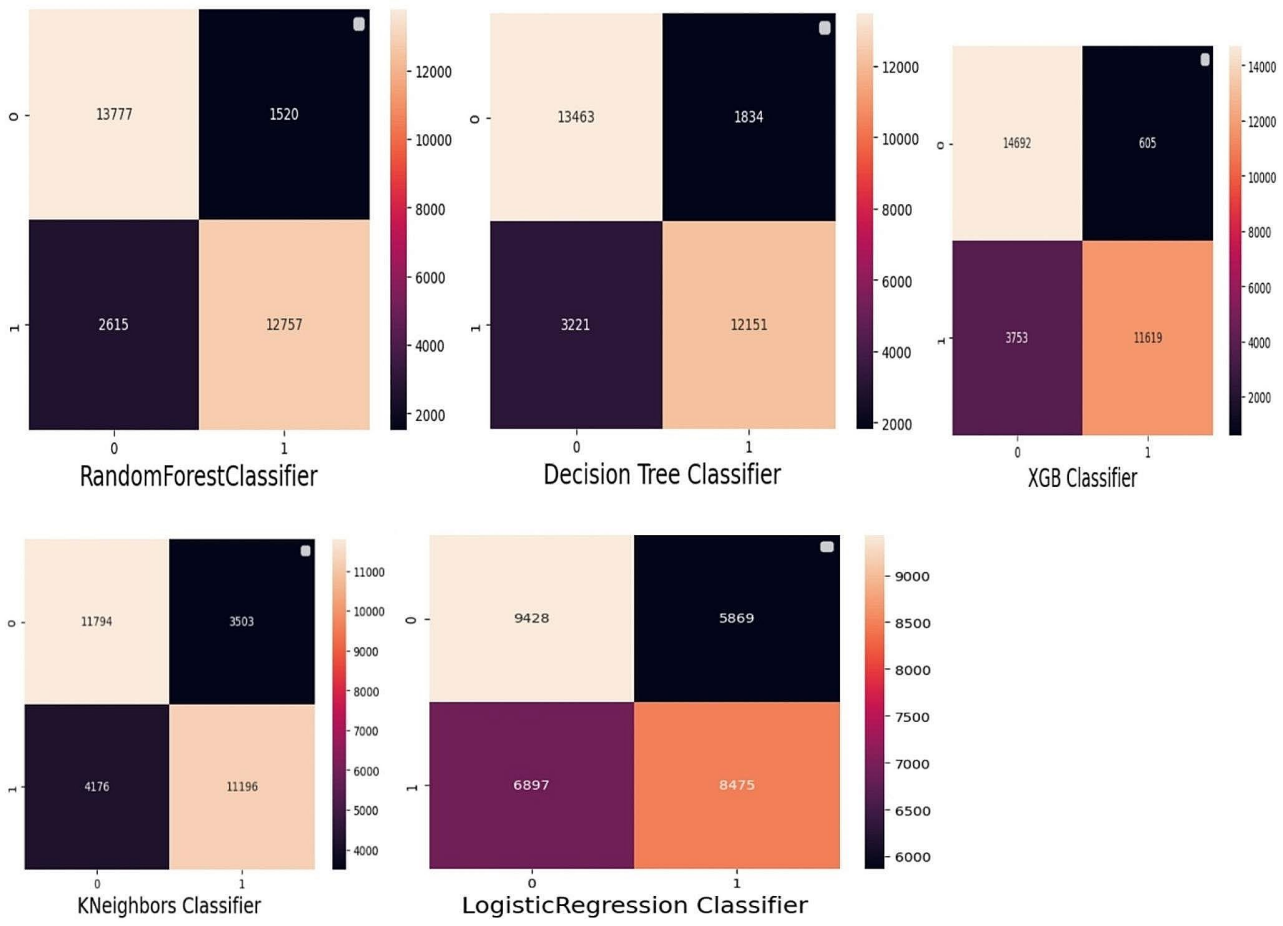
Confusion matrix for proposed

The study compared four different supervised machine learning classification methods to verify the superiority of one of the proposed methods. The diarrhea disease status outcome prediction of Random Forest, Decision Tree (DT), GB (Gradient Boosting), K-Nearest Neighbor, and Logistic Regression experiments were done with the same testing parameters. Since accuracy, AUC, precision, recall, and F-measure are the parameters used to evaluate the performance of the model, and since RF performs the best overall in the proposed model, it was chosen as the top machine-learning algorithm. The outcomes are displayed in (Fig. 4), (Fig. 5), and (Table 3). With an accuracy of 86.5%, precision of 89%, F-measure of 86%, AUC curve of 92.7%, and recall of 82%, random forest is the best classifier in this study.

**Fig. 6:**
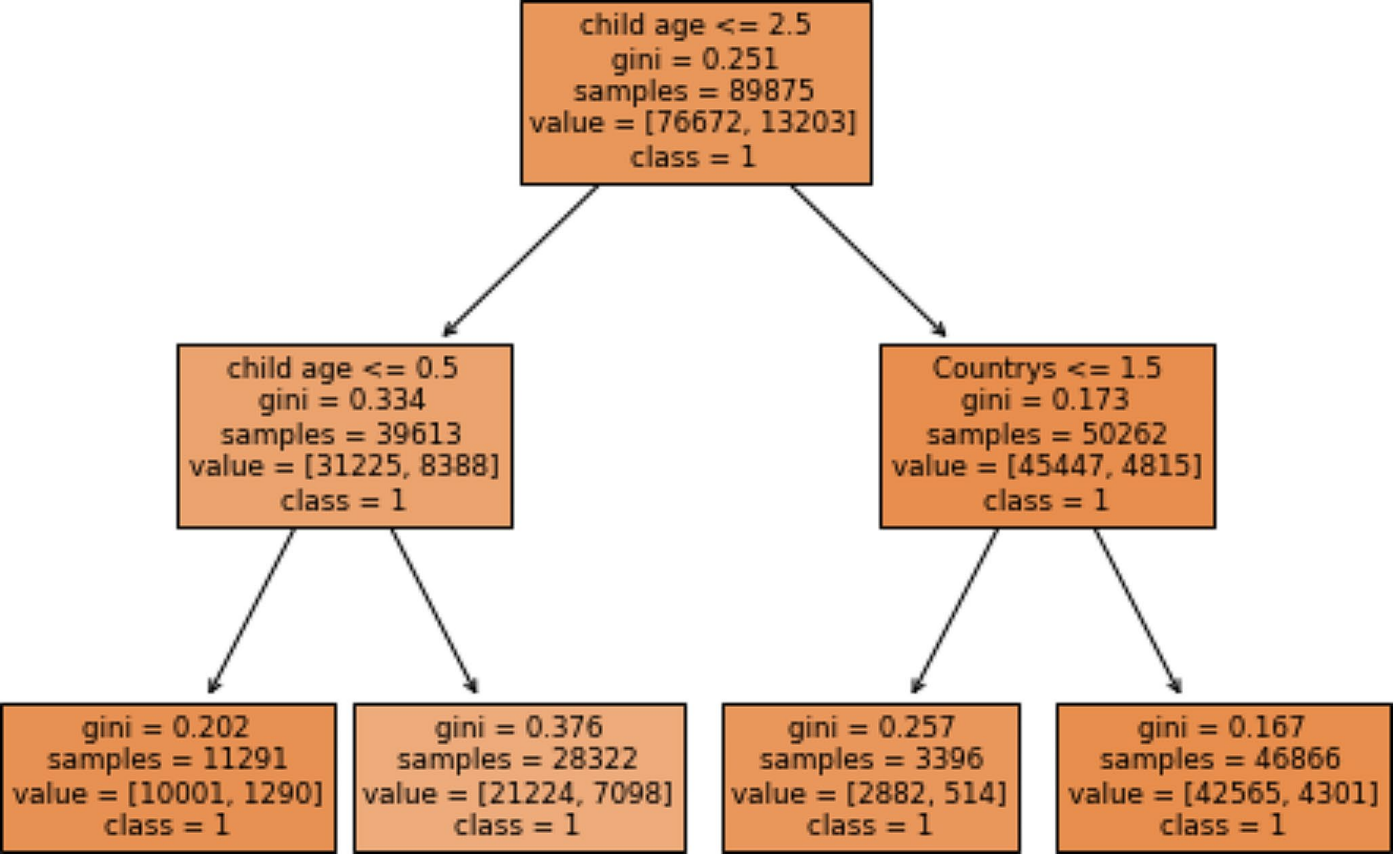
important rules that can be generated from the random forest

Furthermore, the random forest had a high true positive rate of 84%, a false positive rate of 10.6%, a true negative rate of 89.3%, and a false negative rate of 0.16%. The error rate discovery was 0.135%, and the AUC curve was high, 92%, as shown in Fig. 6.

**Table 3:** Accuracy, Precision, Recall and F-measure for the machine learning algorithms

**Machine learning model**					
**Metrics**	**GB**	**DT**	**RF**	**LR**	**KN**
**Accuracy**	85.7%	83.5%	86.5%	58.37%	58.2%
**Precision**	95%	86%	89%	58%	76%
**Recall**	75%	79%	82%	54%	72%
**F-measure**	84%	82%	86%	56%	74%
**AUC**	92%	87%	92%	60%	83%

### Important Rules that can be Generated from the Predictive Model

In this study, we generate important rules by using the best-performing or selected model (gradient boosting with all selected determinants) for the status of diarrhea disease among under-five children in east Africa. As shown in (Fig. 6), max-depth = 2 and random states = 0. The rules listed below have been verified by experts in the field employed by University of Gondar referral hospitals. We suggested that these guidelines are essential for formulating strategies and policies to stop or manage diarrheal illness among children under five in East Africa.

#### Rule 1

IF children age ==’7–12 months’ and countries ==’Kenya’ and wealth status = = poor’ and mothers educational status = = no ‘education ‘and mother age ==’15 = 24’ and source of drinking water=’improved’ and number of under five children = = gather than two and immunization status==’ partially and media exposure = = no and timing of breast feeding ==’ 0 to 1 h’ and mothers working status ==’ not working’ and types of toilets ==’unimproved’ and twins status ==’no ‘THEN diarrhea disease status ==’YES’.

**Rule 2:If children age ==’**25–59 month’ and countries ‘**Mozambique’** and wealth status ==’middle’ and mothers educational status = = primary ‘education ‘and mother age ==’35 = 49’ and source of drinking water = = improved’ and number of under five children ==’one’ and immunization status ==’ fully’ and media exposure = = yes’ and timing of breast feeding ==’ 0 to 1 h’ and mothers working status ==’ working’ and types of toilet =’improved’ and twins status ==’no’ THEN diarrhea disease status =’No’.

## Discussion

This study used the classification machine learning method to compare, identify, and help recognize specific risk factors related to diarrhea disease among under-five children in east African countries that can be used as intervention targets. When compared to other machine learning classifier models such as the RF, DT, GB, KN, and logistic regression, with an accuracy of 86.5%, precision of 89%, F-measure of 86%, AUC curve of 92%, and recall of 82%, random forest is the best classifier in this study. Our results were best with those made in Uganda, which indicated gradient boosting was highly significant for predicting diarrhea disease with an accuracy of 70% [32]. The reason for this research revealed in Uganda is that it used only one DHS data set; however, this study used 12 DHS datasets, so the large datasets increase the performance of the of the machine learning model [33]. And the study conducted in Zimbabwe predicted diarrhea disease. The study revealed that logistic regression was the was the best model, with a prediction accuracy of 85% [28]. It is comparatively good. To our knowledge, no previous studies have demonstrated the benefits of machine learning for predicting diarrhea disease in east Africa. As the feature importance rank identified, children age, countries, wealth status, mothers educational status, mother age, age, and source of drinking water, the number of under-five children immunization status, media exposure, timing of breast feeding, mothers working status, types of toilets, and twin status were the critical predictors of a chance of diarrhea disease in east Africa, according to the Gradient Boosting classification model.

Some of these variables had already proven to be predictors of diarrhea disease in previously published studies. The first important feature of east Africa as a predictor of diarrhea disease was children’s age.

The prevalence of developing diarrheal diseases was higher in children aged 7–12 and 13–23 months compared to children aged 0–6 and 24–59 months. This finding is in agreement with other studies [2, 34]. One of the reasons is that at this age, the immune system weakens and starts complementary feeding like bottle feeding because somehow it has hygiene problems, so it can simply be affected by viruses, bacteria, and parasites.

We found that children who used unimproved toilets were more likely to report having diarrhea disease compared with those who used improved toilets. This could be due to poor hygiene or the luck of washing hands with soap and water before eating. This finding is in agreement with what was found in Ethiopia [35, 36] and Senegal [16].

Inadequate feeding may also make it more vulnerable to diarrhea. In this study, diarrhea is a major health problem in low- and middle-income countries. The findings are supported by similar findings [37–39]. Diarrhea disease was significantly more common in children whose mothers or caregivers had no media exposure than in caregivers who had media exposure. Children with no media exposure had a 15% higher chance of being detected by diarrhea disease. These findings are supported by similar findings in Bangladesh [40]. Mother is aware of these aspects because she has been exposed to the mass media about diarrheal illness and its easy treatment.

## Conclusion and Recommendation

Machine learning approaches can be used to classify certain hidden information that is indiscernible using conventional statistical tools. The findings of the last experiment showed that the gradient boosting model was the most accurate at evaluating risk factors and predicting diarrhea disease among children under five. The important determinants selected by Gradient Boosting were children age, countries, wealth status, mothers educational status, mother age, source of drinking water, number of under-five children immunization status, media exposure, timing of breast feeding, mothers working status, types of toilets, and twin status. Policymakers should consider the research’s findings and develop a plan for decreasing child mortality by diarrhea disease in east African nations based on the variables that have been found to be significant. Despite the intriguing outcome, more work has to be done using different kinds of approaches with different parameters. It is also advised that moms be fully aware of sanitation and feeding their children. Furthermore, moms who lack education should be made aware of the nature of diarrhea in children.

### Strength and Limitations

In this study, the main strength is the recent DHS dataset, and the dataset was integrated from 12 countries DHS databases, which are the same data collection tools, and this study has such a huge sample size. Nevertheless, since DHS data collection is self-reported, there may have been some information bias added, which limits this study.

## Data Availability

The datasets used in this analysis are publicly available in the DHS Program repository. After we submitted the research question, we were granted permission to access the data via the measure DHS program online request form on the website (https://dhsprogram.com/).

## Abbreviations

DHS: Demographic and Health Surveys
DT: Decision Trees
FR: Random Forests
ML: Machine Learning
GB: Gradient Boosting
LR: Logistic Regression
